# Effectiveness of couple education and counseling on uptake of cervical cancer screening among women in Southern Ethiopia: a cluster randomized trial

**DOI:** 10.1038/s41598-024-61988-2

**Published:** 2024-05-31

**Authors:** Samuel Yohannes Ayanto, Tefera Belachew, Muluemebet Abera Wordofa

**Affiliations:** 1 Department of Population and Family Health, Jimma University, Jimma, Ethiopia; 2 Department of Nutrition and Dietetics, Jimma University, Jimma, Ethiopia

**Keywords:** Cervical cancer screening, Couple education, Effectiveness, Ethiopia, Cancer, Health care

## Abstract

Cervical cancer is a major public health problem worldwide. Women die of the disease due to low early screening practices and its detection at advanced stages particularly in developing countries. Therefore, this study aimed to determine the effectiveness of couple education and counseling on the uptake of cervical screening among women. The study employed random allocation of 16 clusters into two study arms. A total of 288 women participated in the study. Structured home-based education and counseling were provided to the intervention group, while the control group received standard of care. Surveys were completed at baseline and end line. This study demonstrated that the proportion of women who had been aware of cervical cancer and screening grew from 22.9 to 100%, participants’ mean knowledge scores showed improvement from 3.18 to 11.99, and cervical screening uptake increased from 2.1% to 72.5% in the intervention group (p < 0.001). Also, the difference in differences of screening uptake between the study groups was statistically significant (p = 0.021).The study shows the effectiveness of the intervention package in improving the uptake of cervical screening in the study setting. Therefore, we recommend that creating awareness, increasing knowledge, and improving women’s perceptions through structured home-based couple education and counseling is important to improve cervical screening uptake among the target women.

## Introduction

Cervical cancer develops as a result of Human Papilloma Virus (HPV) infection. Nearly all women who experience sexual intercourse are infected with the virus in their lifetime but most of these infections spontaneously disappear within 2 years^1^. However, nearly 12% of the infections persist and evolve to precancerous lesions or advanced stages, when detection and treatment is delayed^2^. The extended course, 10 to 20 years, in the progression of HPV infection provides a good opportunity to execute effective early screening and detection programs to prevent the development of cervical cancer^1^.

An estimated 604 127 cervical cancer cases and 341 831 deaths occurred globally in 2020 among women. Cervical cancer incidence was three times and mortality rates were six times higher in countries with low Human Development Index (HDI) compared with very high HDI in the year^3^. Also, in 2018, it occurred approximately in 570 000 women and 311 000 women died globally. Moreover, in rich nations cervical cancer incidence and mortality were two to four times lower than what was in resource scarce nations in the year^4^. These figures show that the burden of cervical cancer among women in less developed regions of the world had been disproportionately high^1^.

There had been larger proportions of morbidity and mortality in Sub-Saharan African countries in 2018 where, Eastern Africa shared the highest burden of the disease in the year^5^. Also cervical infection with HPV is highly prevalent in Sub-Saharan Africa (SSA)^6^. From the global mortality projections made by World Health Organization (WHO) for the year 2030, 98% of deaths will take place in low income countries, with highest mortality in SSA^7^.

According to a summary report of Human Papilloma virus and related diseases in Ethiopia, 36.9 million female population is at risk for cervical cancer. Annually, 7445 new women develop cervical cancer disease and 5338 die from the disease in the country^8^. Also, there were 6047 new cases of cervical cancer with age specific incidence rate of 22% in the country in 2015^9^. In a hospital based study conducted in Addis Ababa, 23.5% of the study participants were screened positive for VIA test^10^ which could have progressed to cervical cancer if it went unnoticed. Cervical cancer is the second-most common cause of female cancer and the second leading cause of female cancer deaths in Ethiopia^8^.

Evidence shows that the magnitude of cervical cancer incidence and mortality has been substantially reduced in developed countries during the last decades. This achievement has been attributed to the execution of screening packages for the timely detection of precancerous cervical lesions and HPV infection and availability of better treatment alternatives^11^. But, in low- and middle income nations where availability of screening and treatment services are constrained, it remained an important public health concern^5^.

In Ethiopia, cervical screening has been made widely available at public healthcare facilities after the development of comprehensive cervical cancer prevention and control guideline in 2015^12^. Before the implementation of this guideline, age-eligible women ever received screening in Ethiopia was nearly 1%^13^. Recently, an uptake of 9.9% –15.5% have been reported in selected population groups ^14–16^ . But this achievement was far away from 80% target set nationally for the 30–49 years women by 2020^17^ that demands urgent public health actions to avert this low uptake of cervical screening among eligible women.

Different studies suggest the importance of male involvement or couple interventions in the uptake of maternal services by target women as males’ approval and encouragement for their use is an important determinant factor^18,19^. Moreover, studies involving effectiveness of locally adapted couple based education intervention options haven’t been conducted among women to increase screening uptake. Our research, therefore, was intended to test the effectiveness of home based structured couple education and counseling on the uptake of cervical cancer screening among women in Southern Ethiopia.

## Methods

### Study context and period

This two arm parallel cluster randomized trial was conducted in two zones of Southern people regional state of Ethiopia from February to September 2022. Two groups of women were compared where the intervention group received home based structured couple education and counseling while the control group received the usual standard of care.

### The population, intervention, comparison, outcome and time frame

The key parameters of description for this trial are the population, the intervention, the comparison, the outcome and the time frame. The population included in the trial was women aged 30–49 years. This population was randomly assigned in to intervention and control groups at cluster level for comparison of the outcome measures. The intervention group was exposed to home based couple education and counseling while the control group received standard of care. The primary outcome of the trial was the completion of cervical cancer screening in 6 months. This 6 months period refers to the overall trial period of the study.

### Participants’ eligibility and recruitment

**Figure 1:**
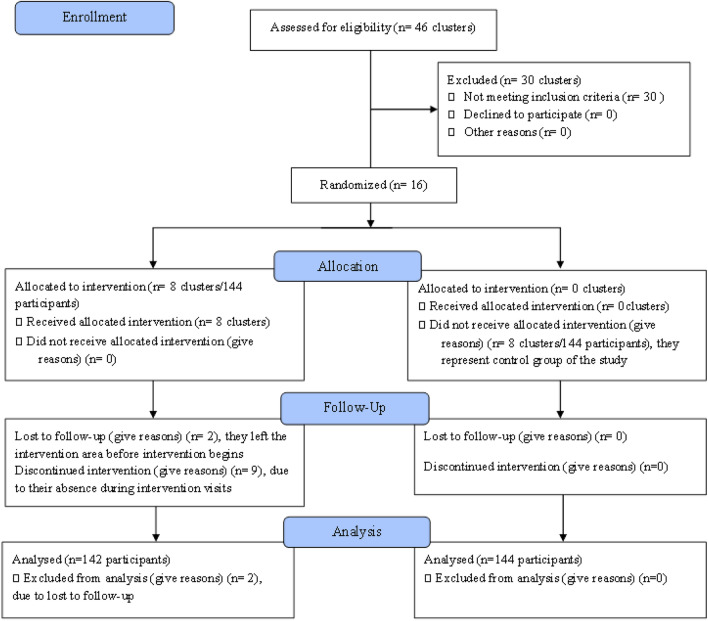
Consort flow diagram.

Two study districts were identified from Hadiya and Kembata-Tembaro zones, one from each, based on the availability of cervical cancer screening program. All the clusters found in the districts were listed. Then, sixteen geographically non-neighboring clusters were recruited from the existing clusters within the two study districts leaving at least one cluster between clusters included in the study to build a buffer zone to prevent information contamination. The selected clusters were stratified by district level and randomly allocated in to intervention and control groups using block randomization techniques to ensure eight clusters in the intervention arm and eight clusters in the control arm. The overall cluster allocation and participant recruitment have been depicted in the Consort Flow Diagram (Fig. 1).

The eligible women for cervical cancer screening according to the Ethiopian national cervical cancer prevention and control guideline (30–49 years)^12^ were targets of the study and those who satisfied specific criteria for the study had been identified through census and health post records. Then, we applied simple random sampling technique to select equal number of study participants from each cluster included in each arm. The participants were recruited to the study in February 2022.

Health extension workers conducted the identification of the participants’ from the selected clusters for the data collection purpose and intervention delivery activities. The identified women at each arm were reached out by health extension workers and the data collectors at their home. Data collectors interviewed each participant after she consented to complete a baseline survey. After the completion of the baseline data, health extension worker of the respective cluster provided the proposed intervention. Finally, the same assessors completed the end line survey at 6 months. The outcome assessors were made blind for the intervention.

### Cluster randomization

Four blocks of four clusters were created and the clusters in each block were randomly assigned to intervention and control arms according to the randomization sequence evident by the selected permutation for each block. Consequently, eight clusters were assigned to the intervention arm and eight clusters to the control arm maintaining 1:1 allocation ratio. The statistician conducted random allocation of the clusters and he was made unaware of which group of clusters will receive the proposed intervention and which will receive the usual standard of care. This was achieved by representing the study arms and clusters by confidential codes.

### Intervention description

Home based brochure assisted couple education and counseling was delivered to women in the intervention group followed by referral services to nearby cervical cancer screening facilities. Participants in the intervention arm received education and counseling at three contact points during the intervention period of which the second contact was designed to deliver the intervention for both the woman and her husband. The woman and her husband were educated about cervical cancer and its screening and counseled on the importance of screening for cervical cancer. The husband was also counseled on the importance of providing support and encouragement to his wife. Additionally, the woman was provided with the educational material during the initial visit for further reading and offered referral slip during the last visit. The educational and counseling brochure was prepared to address susceptibility to cervical cancer, its seriousness, the benefits of screening and barriers to screening to further build cues to action and woman’s self-efficacy using the health belief model. The intervention was designed to bridge the knowledge gap among women and positively influence their belief system related to cervical cancer and its screening which in effect would bring positive behavior among women to demonstrate screening performance.

### Implementation of the intervention

The trial period was organized in two phases and lasted for 6 months. In phase one, the proposed intervention package was actively delivered for the first 3 months to each woman in a monthly base at the home environment. But, in phase two participants did not receive the intervention for the last three months of the trial period but left without the intervention to provide a time for rehearsal & translation of knowledge in to practice. Each woman in the intervention clusters was physically visited three times at her residential home. During the initial contact, in average, a 45 min brochure guided education & counseling session was held with the woman in her home environment to convey information on cervical cancer & its screening and counsel her to encourage screening uptake. At the end of the session, discussion was held with the woman to address any questions and concerns. The woman was provided with the educational brochure for further reading in her convenience at least once per week by herself or to be read by any literate person within the family or neighborhood.

The second visit was made to each woman one month after the initial visit based on the appointment to re-emphasize important points of the material and make further encouragement for screening. During this visit both the woman and her husband together received key messages on cervical cancer and the importance of its screening. The husband was also counseled on the way how the woman receives his support and encouragement to get screened for cervical cancer. During the third contact, key messages about cervical cancer and its screening were highlighted and any persistent concerns related to cervical cancer and its screening were addressed. Also, a formal referral slip was granted during this visit for free screening services available in the nearby health facilities.

### Standard of care

The control group did not receive any innovative health care services in this period but received standard of care as described in details in the published protocol^20^. Generally, the usual standards of care provided at home environment involved preventive and promotive health care services. Health extension workers provide such services to households at home level with the objective to reinforce behavioral change among household members on the use of care services including disease screening included in the health extension packages. These services are packaged under four major program areas such as disease prevention and control; family health; personal hygiene and environmental sanitation; and health education and communication. Finally, the control group has been exposed to the innovative intervention after the trial period through the routine service delivery schemes.

### Intervention summary and compliance parameter

Participants in the intervention arm were visited repetitively in a case of their absence at home to improve their compliance to full intervention package. In spite of such efforts, few of the participants failed to fully comply with the proposed intervention as per the protocol. A compliance checklist was used to track the level of women’s compliance to our proposed intervention package to account for it during data analysis. We have indicated a summarized version of the intervention description and level of compliance in the table (Table 1).

**Table 1:** Summary of the intervention description and level of compliance to the intervention package among the study participants, Southern Ethiopia, 2022.

Type or nature of the intervention	Dosage	Frequency	Duration	Compliance parameter
Brochure guided couple education & counseling	45 min	Three rounds	Three months	92.4% full compliance to the intervention package
Participants were provided with referral slip to link them to the nearby screening facilities at the end of the third round of Brochure guided couple education & counseling				

### Primary outcome

The primary outcome of this study was the completion of cervical cancer screening test within 6 months of the the trial period. Participants were interviewed using the survey questionnaire and also tracked via health records for completing the screening test. We also assessed the progress of participants’ knowledge and attitude by measuring their knowledge and attitude scores at baseline and end line.

### Sample size

We identified current cervical cancer screening uptake from the existing literatures to be 15.5% among age eligible women^16^ and considered it as a baseline proportion that was assumed for the control arm. We anticipated an absolute increase of 20% in cervical cancer uptake from the baseline that produces the expected end line proportions of cervical cancer screening for the intervention arm to be (15.5% + 20%) 35.5%. Setting the power of the study at 80%, significance level for one-tailed test at 5%, and considering the design effect of 2 to adjust for the loss of variability due to clustering and 5% as a compensation for incomplete and non-response rates, we arrived at a total of 288 study participants.

### Data collection tool and methods

We prepared and used structured questionnaire to collect the data. The tool was prepared from different literature sources and pretested and validated by taking 5% of the sample size before actual research data collection. Participants were interviewed face to face at their home by trained health professionals to complete the baseline and end line survey. Participants were measured for socio-demographic characteristics, knowledge, attitudes, and cervical cancer screening uptake. The authors had no access to information that could identify individual participants during or after data collection.

### Data management and analysis

Specific participant and cluster codes were given for each of the completed questionnaire. The data were entered and edited using Epi info version 7.2.4.0 and transported to SPSS version 27 to carry out the desired statistical analysis. Baseline screening proportions with 95% confidence interval were computed for both arms. The groups were assessed at base line for any statistical differences with respect to baseline characteristics using chi-square test for categorical variables and independent sample t-tests for continuous variables. A one-sided *P* value was used to determine statistical significance of the tests.

At the end line, we calculated proportions of women screened, their knowledge and perception scores, and compared the before and after intervention values for each arm. We used paired sample t-test for continuous variables and paired proportions test for categorical variables. Then, the independent-sample t-test was performed to determine the presence of statistically significant difference in the participants’ knowledge and attitude between the study arms. The chi-square test was carried out to compare categorical variables between the arms. The Likelihood ratio value was used for tables bigger than 2 × 2 instead of Pearson Chi-square when the assumptions were violated. The results were computed in terms of the test statistic, confidence intervals and *p*-values.

Finally we analyzed the difference of differences of cervical screening performance to examine the net effect of the intervention and carried out Generalized Estimating Equations analysis technique to identify the independent determinants of the uptake of cervical screening services. The effect size of the intervention, confidence interval and associated *P*-value were determined during the analysis. Then, 95% confidence intervals of the estimates were determined by considering the sample estimates and the margin of error of the estimate to indicate the interval limits. The methodological details of this study have been presented within the trial protocol published elsewhere^20^. All methods were performed in accordance with the relevant guidelines and regulations.

### Validity and reliability of the study

To ensure the quality of the study outcomes we addressed the study validity and reliability using different techniques. The validity of the study has been systematically addressed by demonstrating possible ways to minimize the effect of chance, bias & confounding to help the findings reflect the true effect of the exposure on the occurrence of the desired outcome. Accordingly, this study employed adequate sample size, study power, random assignment of the groups, testing the research instrument, training of the data collectors, and appropriate statistical analysis to control for confounding. Also, the reliability of this study has been ensured by measuring the reliability coefficient of the data collection tool. Those items which scored a reliability coefficient below the acceptable range have been excluded from the analysis. Also, all the methodological details employed in the research design, data collection, and data analysis stages that suit the research purpose have been clearly described to ensure its consistency and help replicate the study by other researchers in other study contexts.

### Ethics approval and consent to participate

This study was approved by the IRB of Jimma University. The IRB of Jimma University Institute of Health approved it with approval number IHRPGn/355 on 16/7/2021. Data collectors explained the objectives of the study to the participants before the interview to obtain consent. Informed consent was obtained from all subjects before data collection through the help of data collectors using the consent format prepared for the research purpose.

## Results

### Participants’ sociodemographic characteristics

During the end-line survey, 142 (98.6%) of the study participants from the intervention group and 144 (100%) of the participants from the control group responded to the questionnaire. All the participants were married women in the age range from 30 to 49 years with the mean age of 38 ± 5.34 and 38 ± 5.11 years for the control group and the intervention group respectively. The difference in the mean age of the participants in the intervention and the control group was not significantly different (*p* = 0.482). There were no statistically significant differences between the intervention and control group in terms of other sociodemographic characteristics like participants’ education, family income, family size, parity level and awareness about cervical cancer and screening at baseline. But the groups were significantly different with respect to health insurance membership (*p* < 0.001) and maternal service use in the last year (*p* = 0.011). More than 70% of the participants in both groups completed primary education (Table 2).

**Table 2:** Baseline characteristics of the study participants, Southern Ethiopia, 2022.

Participants’ characteristics	Categories	Intervention group N = 144 (%)	Control group N = 144 (%)	Test statistics	*P*-value
Age	Intervention vs control	38 (SD = 5.11)	38 (5.34)	t = 0.045 (df = 286)	0.482
Religion	Protestant	110 (76.4)	114 (79.2)	χ2 = 0.724	0.432
	Orthodox	15 (10.4)	15 (10.4)		
	Islam	9 (6.2)	6 (4.2)		
	Others	10 (6.9)	9 (6.2)		
Ethnicity	Hadiya	86 (59.7)	84 (58.3)	LR = 1.96	0.375
	Kembata	57 (39.6)	56 (38.9)		
	Others	1 (0.7)	4 (2.8)		
Education level	No formal education	26 (18.1)	25 (17.4)	LR = 0.453	0.465
	Primary education	101 (70.1)	105 (72.9))		
	Secondary education	14 (9.7)	12 (8.3)		
	Tertiary education	3 (2.1)	2 (1.4)		
Occupation status	Domestic roles	130 (90.3)	136 (94.4)	χ2 = 1.77	0.092
	Others	14 (9.7)	8 (5.6)		
Membership in WDA	Yes	79 (54.9)	72 (50)	χ2 = 0.682	0.205
	No	65 (45.1)	72 (50)		
Health insurance membership	Yes	103 (71.5)	73 (50.7)	χ2 = 13.149	< 0.001
	No	41 (28.5)	71 (49.3)		
Maternal service use last year	Yes	129 (89.6)	115 (79.9)	χ2 = 5.258	0.011
	No	15 (10.4)	29 (20.1)		
Monthly family income	1st quartile	44 (30.6)	41 (28.5)	χ2 = 2.522	0.236
	2nd quartile	34 (23.6)	25 (17.4)		
	3rd quartile	31 (21.5)	38 (26.4)		
	4th quartile	35 (24.3)	40 (27.8)		

LR = likelihood ratio; WDA = Women's development army.

### Participants’ knowledge, perceptions and screening uptake at baseline

The awareness level of the participants in both the intervention and control groups regarding cervical cancer and screening was low at baseline in that 22.9% and 20.1% of the participants ever heard of cervical cancer in the intervention and control groups respectively. The difference in awareness level was not statistically significant. Only 2.1% of the participants in the intervention and 0.7% in the control group have had cervical screening experiences at baseline. More than 3/4^th^ of the participants in both the intervention and control groups had poor knowledge regarding cervical cancer and screening. The mean and standard deviation of knowledge scores for the intervention group was 3.18 (SD = 4.47) whereas, the control group had mean score of 3.44 (SD = 4.74). These differences are not statistically significant.

The perceived severity score for the disease and perceived benefits score of screening were higher in both groups at baseline. However, the difference was not statistically significant. For example, the majority of the participants in the intervention (62.5%) and control (65.3%) groups had mean and above score in perceived severity items. Also, participants who earned mean and above score in perceived benefit items were 61.8% and 57.6% in the intervention and control group respectively. The mean scores for perceived susceptibility, perceived barriers and readiness for cervical screening were lower in both groups. The study groups were not significantly different in terms of the perception scores (Table 3).

**Table 3:** Participants’ awareness, knowledge of cervical cancer and screening, perceptions, and uptake of cervical screening at baseline, Southern Ethiopia, 2022.

Variable	Intervention	Control	Test statistic	*P*-value
Ever heard of cervical cancer and screening	33 (22.9%)	29 (20.1%)	χ2 = 0.329	0.283
Cervical screening uptake*	3 (2.1%)	1 (0.7%)	Fisher’s exact test	0.311
Mean knowledge score	3.18 (SD = 4.47)	3.44 (SD = 4.74)	t = -0.499 (df = 286)	0.309
Mean perceived susceptibility score	1.61 (SD = 0.56)	1.69 (SD = 0.57)	t = -1.218 (df = 286)	0.112
Mean perceived severity score	2.47 (SD = 0.49)	2.5 (SD = 0.51)	t = -0.444 (df = 286)	0.329
Mean perceived benefit score	2.42 (SD = 0.58)	2.5 (SD = 0.54)	t = -1.226(df = 286)	0.111
Mean perceived barriers score	1.76 (SD = 0.57)	1.79 (SD = 0.51)	t = -0.438 (df = 282)	0.331
Mean readiness score	1.49 (SD = 0.80)	1.56 (SD = 0.76)	t = -0.832 (df = 286)	0.203

*Primary outcome; (SD = standard deviation).

### Participants’ knowledge, perceptions and screening uptake at endline

At the end-line, among participants in the intervention group, the awareness of cervical cancer and screening became higher; and the knowledge and perception levels were considerably increased. However, the control group remained lower in terms of awareness, knowledge and perception scores. Also, the proportion of mothers who received screening has been improved among the intervention group whereas, screening performance remained low in the control group. The difference between the intervention and the control group with regard to the above parameters was statistically significant (*p* < 0.05) (Table 4).

**Table 4:** Participants’ awareness, knowledge of cervical cancer and screening, perceptions, and uptake of cervical screening post intervention, Southern Ethiopia, 2022.

Variable	Intervention group	Control group	Test statistic	*P*-value
Ever heard of cervical cancer and screening	142 (100%)	32 (22.2%)	χ2 = 181.54	*P* < 0.001
Cervical screening uptake*	103 (72.5%)	2 (1.4%)	χ2 = 155.76	*P* < 0.001
Mean knowledge score	11.99 (SD = 2.64)	3.45 (SD = 4.70)	t = 18.97(df = 226)	*P* < 0.001
Mean perceived susceptibility score	2.03 (SD = 0.91)	1.72 (SD = 0.53)	t = 3.55 (df = 227)	*P* < 0.001
Mean perceived severity score	2.66 (SD = 0.64)	2.50 (SD = 0.34)	t = 2.6 (df = 213)	*P* = 0.005
Mean perceived benefit score	2.72 (SD = 0.55)	2.47 (SD = 0.48)	t = 4.02 (df = 284)	*P* < 0.001
Mean perceived barriers score	1.06 (SD = 0.19)	1.82 (SD = 0.54)	t = -15.87 (df = 179)	*P* < 0.001
Mean readiness score	2.49(SD = 0.85)	1.59 (SD = 0.84)	t = 9.04 (df = 284)	*P* < 0.001

*Primary outcome.

### Effectiveness of the intervention

The Brochure assisted home based couple education and counseling intervention had brought statistically significant effect on the knowledge, perceptions and screening uptake among the study participants. The mean knowledge and perceptions scores showed statistically significant improvement with regard to cervical cancer and screening in the intervention group. The perception constructs of the health belief model demonstrated statistically significant improvement among the participants in the intervention group. Consequently, the perception of the study participants in the intervention group was improved in terms of cervical cancer susceptibility, severity and benefits of cervical screening. Also, the perceived barriers to screening showed statistically significant decrement among participants in the intervention group. Moreover, the proportion of women who received cervical screening showed a marked improvement among the participants in the intervention group. However, there was no statistically significant difference in knowledge, perceptions and screening performance among the participants in the control group between baseline and end-line (Table 5).

**Table 5:** The effect of home based Brochure assisted couple education and counseling on cervical screening use among study participants, Southern Ethiopia, 2022.

Variables	Intervention group					Control group				
	Baseline (n = 144)	Post intervention (n = 142)	Difference	Test statistic	*P*-value	Beginning (n = 144)	End (n = 144)	Difference	Test statistic	*P*-value
Ever heard of cervical cancer & screening (proportions)	0.229	1	0.771	0.768, 95%CI (0.691, 0.844)	< 0.001	0.201	0.222	0.021	0.021, 95%CI (-0.009, 0.051)	0.124
Cervical screening uptake* (proportions)	0.021	0.725	0.704	0.704, 95%CI (0.622, 0.786)	< 0.001	0.007	0.014	0.007	0.007 95%CI (-0.014, 0.027)	0.50
Mean knowledge score	3.18 (SD = 4.47)	11.99 (SD = 2.64)	8.767	t = 19.944 (df = 141)	< 0.001	3.44 (SD = 4.74)	3.45 (SD = 4.70)	0.0069	t = 0.199 (df = 143)	0.421
Perceived susceptibility score	1.606 (SD = 0.55)	2.028 (SD = 0.907)	0.423	t = 4.669 (df = 141)	< 0.001	1.69 (SD = 0.57)	1.742 (SD = 0.53)	0.023	t = 0.785 (df = 143)	0.217
Perceived severity score	2.47 (SD = 0.49)	2.66 (SD = 0.64)	0.1907	t = 2.831 (df = 141)	0.0025	2.5 (SD = 0.51)	2.50 (SD = 0.34)	0.0081	t = 0.224 (df = 143)	0.412
Perceived benefit score	2.42 (SD = 0.58)	2.72 (SD = 0.55)	0.3068	t = 4.422 (df = 141)	< 0.001	2.5 (SD = 0.54)	2.47 (SD = 0.48)	-0.0225	t = -0.528 (df = 143)	0.299
Perceived barriers score	1.76 (SD = 0.57)	1.06 (SD = 0.19)	-0.7012	t = 14.383 (df = 141)	< 0.001	1.79 (SD = 0.51)	1.82 (SD = 0.54)	0.0258	t = 0.711 (df = 143)	0.239
Readiness score	1.49 (SD = 0.80)	2.49(SD = 0.85)	1.014	t = 9.421 (df = 141)	< 0.001	1.56 (SD = 0.76)	1.59 (SD = 0.84)	0.0278	t = 0.533 (df = 143)	0.298

*Primary outcome.

The proportion of participants in the intervention group with good knowledge score on cervical cancer and screening grew from 32.6% to 98.6%. On the other hand, the proportion of participants with good knowledge in the control group progressed only from 31.9% to 33.3%. Also, the proportion of participants with a good perception about barriers to cervical cancer screening was improved from 49.3% to 98.6% in the intervention group, whereas it was reduced from 45.8% to 41.7% in the control group. Similarly, the percentage of women who had good score in perceived benefit of screening in the intervention group grew from 61.8% at baseline to 80.6% at end-line but in the control group it rose only from 57.6% to 59%. Moreover, the mean score for risk perception showed significant improvement among participants in the intervention group unlike that of the control group.

The proportion of women who have been informed about cervical cancer and screening rose from 22.9% at baseline to 100% after the intervention with the absolute increase of 77.1% which is statistically significant (*p* < 0.001). The progress in the awareness level between the baseline and endline study was insignificant and only 2.1% in the control group (*p* = 0.124). Similarly, the cervical screening uptake among participants in the intervention group showed statistically significant increase from 2.1% at baseline to 72.5% after the intervention with 70.4% absolute increment (*p* < 0.001), whereas the participants in the control group demonstrated poor progress in terms of cervical screening uptake during the study period with only 0.7% change (*p* = 0.5) (Table 5).

As identified by the study participants during the baseline study, the major barrier, 71.5% in the intervention group and 78.5% in the control group, to the uptake of cervical screening was lack of awareness about cervical cancer and screening. However, during the endline survey, lack of awareness was not reported as a reason for not receiving cervical screening among the participants in the intervention group.

### Difference in differences analysis

**Figure 2:**
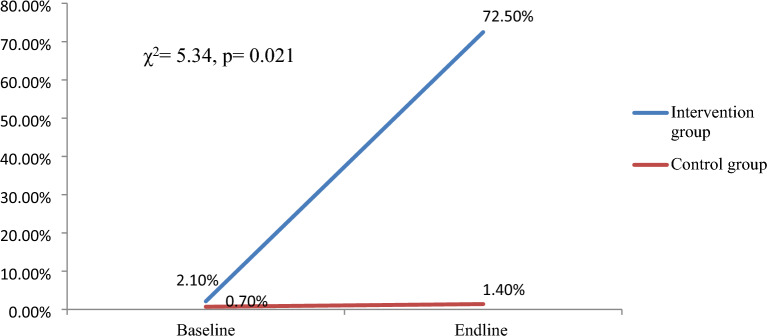
Cervical cancer screening utilization trend among the study participants at baseline and end-line between the study groups, Southern Ethiopia, 2022.

We performed graphical representation and chi-square for trend analysis of the screening proportions of the groups using analysis for linear trends in proportions in EpiInfo StatCalc to determine the presence of statistically significant difference in screening performances. Accordingly, the difference in differences (0.697, *p* = 0.021) in screening performances between the groups is statistically significant (Fig. 2).

### Dose response analysis

All the participants within the intervention group did not comply with our intervention package fully but, over 92% of the participants in the intervention group received the intervention package fully, and less than 8% did not. The relation between intervention intensity and uptake of cervical screening was cross tabulated using linear trend chi-square test. There is a linear by linear association between intervention frequency and cervical screening uptake among the study participants. Consequently, as the dose of the intervention went from none to three, the likelihood of using cervical cancer screening increased significantly (χ^2^ = 169, df = 1, *p* < 0.001) which implies the importance of repeated exposure to the proposed intervention of interest to bring about the required improvement in the uptake of cervical screening among the participants.

### Factors associated with screening uptake

In the multivariable generalized estimating equations, intervention status and husband’s education were statistically significantly associated with cervical screening use among the study participants. Consequently, participants with no formal education of their husband were 93% less likely to use cervical screening as compared to those whose husband attained tertiary education [(AOR: 0.066; 95% CI: (0.010, 0.446)]. Also, those who received the intervention were nearly 43 times more likely to use cervical screening when compared to those who did not receive the intervention [(AOR: 42.68; 95% CI: (2.21, 822.8)] (Table 6).

**Table 6:** Multivariable generalized estimating equations for effect of couple education & counseling on cervical screening use among study participants, Southern Ethiopia, 2022.

Variable	Variable category	n	B	Adjusted OR 95% CI	*P* value
Education of the husband	No education	29	-3.00	0.066 (0.010, 0.446)	0.005
	1° education	165	-1.865	0.199 (0.059, 0.671)	0.009
	2° education	80	-1.643	0.021 (0.239, 0.071)	0.021
	3° education	12		1	0.000
Received Intervention	Yes	140	3.69	42.683 (2.21, 82.3)	0.013
	No	146		1	0.000

Parameters were adjusted for age at marriage, knowledge of someone with cervical cancer, membership in health insurance, husband’s participation in community activities, maternal services use, family members received health services previous year, knowledge score, and perceived barrier score.

## Discussion

This study involved home based structured couple education and counseling to improve the knowledge, perceptions and uptake of cervical screening among women who were eligible for screening. As far as our knowledge is concerned, this is the first effectiveness cluster randomized controlled study regarding cervical cancer screening uptake in Ethiopia. At the outset of the study, baseline assessments revealed low levels of cervical cancer screening uptake, awareness, and knowledge among women in the study settings. Accordingly, it was 2.1% and 0.7% for screening uptake, 22.9% and 20.1% for awareness level and 3.18 ± 4.47 and 3.44 ± 4.74 for knowledge score at baseline in the intervention and control group respectively. However, the proportion of women with good knowledge score, perception scores and cervical screening uptake increased substantially from baseline to end-line in the intervention group. The proportion of women who underwent screening in the intervention group showed considerable increase, 70.4% absolute increase, among women in all age groups particularly among women above age 40 years.

The proportion of the study participants who have heard information regarding cervical cancer and screening at baseline was 22.9% in the intervention group and 20.1% in the control group. This lower figures show that, lack of awareness about cervical cancer and screening has been the main reason for not receiving screening services evidenced by low screening uptake among the study participants at baseline. Other studies showed similar findings and reported low awareness level among women as the main reason for low uptake of cervical screening^21,22^. However, at the end-line survey, after the intervention was provided, the proportion of women who had been informed about cervical cancer and screening demonstrated a significant improvement in the intervention group. A study conducted in Nigeria to test the effectiveness of education intervention produced similar finding regarding awareness level^22^. These findings emphasize the need for designing policy directions that promote awareness creation interventions to improve implementation of the screening programs.

The proportion of women with good knowledge score at baseline in the control and intervention group was 32.6% and 31.9% respectively. Nearly similar finding (30.3%) was observed among women in a study conducted in Finoteselam Ethiopia^23^. On the other hand, the overall pooled prevalence of good knowledge about cervical cancer in Ethiopia is 43%^24^ which is nearly in alignment with our findings. The mean knowledge score among the participants significantly increased among women in the intervention group (*p* < 0.001). Also, the proportion of women with good knowledge considerably increased from 32.6% at baseline to 98.6% at endline whereas, the change remained poor among the control group. Similarly, a very brief educational intervention in rural Kenya resulted significant changes in the knowledge of the study participants in the intervention group^25^. This indicates that structured educational intervention efforts play key role in improving the knowledge level of the target group that imply the importance of program activities that help improve cervical cancer and screening knowledge of the target group to establish causal pathway to screening practices.

All the perception measures of HBM have shown statistically significant changes in their mean score between before and after the intervention when compared using paired samples t-tests. Consequently, there was a statistically significant difference between before and after the intervention scores for perceived susceptibility (*p* < 0.001), perceived seriousness (*p* = 0.003), perceived benefits (*p* < 0.001), and perceived barriers (*p* < 0.001) in the intervention group. This finding is in accordance with the findings from other studies even though the educational methodologies and duration of the intervention differ^22,26^. Similarly, a study conducted in South Asia and Turkey reported a significant improvement in perceived benefits and reduction in perceived barriers among women in the intervention group^27,28^. These findings suggest the significance of designing and integrating interventions, which positively influence women’s perceptions towards screening behavior, in the implementation of screening programs.

As the baseline study has shown, screening uptake was low among the study participants in our study where only 0.7% in the control group and 2.1% in the intervention group received screening in the last five years before the study. Studies have shown that screening proportion for cervical cancer among women in Ethiopia is low, less than 10%^29–32^. However, studies in urban areas of the country demonstrated relatively higher prevalence of screening uptake among women^33,34^ but still lower achievements than what is expected nationally. A systematic review and meta-analysis revealed that the pooled prevalence of screening uptake among age eligible participants in Ethiopia is 13.46%^35^ which is relatively higher than what was observed in our baseline study due to the fact that most of the included studies in meta-analysis involved participants who were positive for Human Immune-deficiency Virus and living in urban settings. In a quasi-experimental study conducted in Nigeria, only 1.1% of the participants had received screening during baseline study in both the control and intervention group^21^. On the other hand, studies revealed a relatively higher proportion (43.48%) in the uptake of cervical cancer screening as seen in Cameroon^36^ even though the pooled prevalence for cervical screening uptake in SSA was determined to be 12.87%^37^. All the above findings illuminated lower levels of cervical cancer screening performances than what is expected of the current disease burden indicating the need for urgent public health interventions to improve screening coverage.

In our study, after the intervention was provided, the screening proportion among women in the intervention group showed statistically significant increase where the screening uptake grew to reach 72.5% (*p* < 0.001). The study conducted among rural communities in Nigeria demonstrated similar findings in that the education intervention significantly improved screening uptake among women in the intervention group^22^. Also, a home based broacher assisted education in Turkey produced a significant improvement in the uptake of cervical cancer screening^38^ However, on the other hand, an effectiveness study on educational intervention conducted on market women in Nigeria did not bring any significant changes in screening uptake^21^ probably because of the context of the intervention in that the study participants being occupied by businesses related matters. Generally speaking, the remarkable increase in the uptake of cervical screening in our study could be due to the fact that the intervention was designed to suit the local context and delivered according to the methodological details contained within the study protocol. This implies that contextually designed and locally structured education and counseling interventions are needed to improve the uptake of cervical cancer screening among the target women.

## Strengths and limitations

This study has its own strengths. It employed randomization techniques to assign the clusters in to the intervention and control arm to get as comparable groups as possible. This effort was vital to distribute confounding variables uniformly across the groups and see the independent effect of the intervention though it came up with its own limitations. Moreover, the study was implemented based on the methodological details and requirements indicated within the published study protocol to ensure the fidelity of the intervention during the trial period.

However, in our study, masking for the intervention was not possible, except for outcome assessors, due to the clustering nature of the units of randomization. However, a buffer zone has been introduced, using clusters that were not part of the study, between the intervention and control clusters to avoid the contamination of intervention messages. The baseline differences suggesting more insurance coverage and maternal service use in the last 1 year among the participants in the intervention group compared to the participants in the control arm could have affected the measurement of the true effect of the intervention package on the primary outcome.

## Conclusion

Structured home based couple education and counseling followed by referral to cervical screening sites showed a remarkable improvement in the uptake of screening services among rural women eligible for cervical screening in Southern Ethiopia. Also, participants’ knowledge on cervical cancer and screening, and their perceptions were significantly improved. The main obstacles for not being screened for cervical cancer were lack of awareness and poor knowledge about cervical cancer and screening. Creating awareness, increasing knowledge and improving women’s perceptions through structured couple education and counseling interventions made cervical screening uptake effective in our study setting. Therefore, scale up of the intervention package to other settings in the health sector could bring the required outcomes in the implementation of cervical screening programs in Ethiopia.

## Data Availability

Data that we used in the research process will be made available through the corresponding author upon requests and appropriate public repositories.

## Abbreviations

HPV: Human papilloma virus
LMICs: Low and middle income countries
SSA: Sub Saharan Africa
WHO: World health organization
